# Reply to Kriventsov, M.A.; Neprelyuk, O.A. Resolvins Revisited: Methodological and Translational Gaps. Comment on “Ghemiș et al. The Involvement of Resolvins in Pathological Mechanisms of Periodontal Disease Associated with Type 2 Diabetes: A Narrative Review. *Int. J. Mol. Sci.* 2024, *25*, 12784”

**DOI:** 10.3390/ijms27073022

**Published:** 2026-03-26

**Authors:** Larisa Ghemiș, Ancuta Goriuc, Ionut Luchian

**Affiliations:** Grigore T. Popa University of Medicine and Pharmacy Iasi, 700115 Iasi, Romaniaionut.luchian@umfiasi.ro (I.L.)

We would like to express our sincere gratitude to the authors of the comment for their careful reading and insightful observations regarding our recently published review, “The Involvement of Resolvins in Pathological Mechanisms of Periodontal Disease Associated with Type 2 Diabetes: A Narrative Review” [1]. We appreciate their valuable contribution to advancing the discussion in this important research field.

First, we would like to clarify that our review was intentionally designed as a **narrative review**, not as a systematic one. Consequently, we adopted an IMRAD-style structure to integrate current knowledge about specialized pro-resolving mediators (SPMs), with an emphasis on resolvins, in the context of the bidirectional relationship between type 2 diabetes mellitus (T2DM) and periodontitis [2,3,4]. As typical for narrative reviews, our objective was to synthesize conceptual and mechanistic insights rather than to conduct an exhaustive quantitative analysis. This aligns with guidelines for narrative reviews.

As is common in narrative reviews, we did not include a numerical description of the number of screened or excluded studies; instead, the methodological section specifies the literature selection process, and all cited works are listed in the reference section. Each reference was chosen based on scientific relevance, novelty, and journal impact, ensuring a balanced and up-to-date evidence base [5,6].

Regarding the **T-series resolvins (RvTs)**, we fully acknowledge their biological relevance, as described by Dalli and colleagues or Mohammad-Rafiei et al. [7,8]. However, at the time when the manuscript was prepared, no preclinical or clinical investigations had directly addressed the involvement of RvTs in the specific setting of type 2 diabetes mellitus and periodontal disease. Existing studies primarily focus on their roles in infection, inflammation, and macrophage efferocytosis, but not in the combined metabolic and periodontal context [7,8,9]. We agree that this represents a highly promising direction for future research, and our group intends to explore it further in upcoming projects.

Since our article is based on presenting up-to-date data on the role of resolvins in these two pathologies, establishing a connection between them without a solid foundation would have been purely speculative in the absence of clear studies.

Once again, we thank the authors for their thoughtful and constructive feedback. We hope that our clarifications contribute to a productive scientific dialogue and that future collaborative efforts will further advance our understanding of SPM biology in chronic inflammatory conditions such as type 2 diabetes and periodontitis.

## Abbreviations

IMRAD-style structure: **I**ntroduction, **M**ethods, **R**esults, and **D**iscussion.
